# Dual-cycle immobilization to reuse both enzyme and support by reblossoming enzyme–inorganic hybrid nanoflowers†

**DOI:** 10.1039/c8ra02051e

**Published:** 2018-04-30

**Authors:** Jianyun Yu, Chenhui Wang, Anming Wang, Ningning Li, Xinxin Chen, Xiaolin Pei, Pengfei Zhang, Stephen Gang Wu

**Affiliations:** College of Materials, Chemistry and Chemical Engineering, Hangzhou Normal University Hangzhou 310014 P. R. China waming@hznu.edu.cn +86-571-28865630 +86-571-28865978; Department of Energy, Environmental and Chemical Engineering, Washington University St. Louis MO 63130 USA

## Abstract

To achieve dual-reuse of both enzyme and support in enzyme immobilization, hybrid nanoflowers (hNFs) were synthesized and crystallized in aqueous solution using calcium phosphate as inorganic component and enzyme as organic component. When hNFs lost their catalytic activity after reuse for times, they underwent dissolution and recrystallization to achieve the dual-cycle of enzyme and support. Six enzymes including papain, bromelain, trypsin, Lipase from Porcine Pancreas (PPL), Lipase from *Thermomyces lanuginosus* (TLL) and Lipase B from *Candida antarctica* (CALB) were chose as model enzymes and the obtained hNFs all presented high catalytic activity and thermal stability. The highest catalytic efficiency (*K*_cat_/*K*_m_) of TLL-hNFs was 38.52 mM^−1^ s^−1^, 21.7 folds than that of free enzyme. Moreover, after heating for 6 h at 70 °C, the residual activities of TLL-hNFs, PPL-hNFs, and CALB-hNFs, were 78.3%, 72.9% and 84.3%, which were 4.57, 2.61 2.35 folds of that of their corresponding free one. Furthermore, the recovery rate of Ca_3_(PO_4_)_2_ were above 95% by recrystallizing the calcium phosphate with fresh enzymes after dissolving the used hNFs and removing the denatured enzyme. The recrystallized hNFs using the recovered phosphate salts and fresh enzymes all gave the consistent catalytic activities. This sustainable dual-cycle method depending on calcium phosphate crystallization, dissolution and recrystallization, was facile and efficient and can also be applied to other enzymes immobilization for industrial biocatalysis.

## Abstract 2

Both enzyme and support can be recycled using dual-cycle immobilization method by reblossoming the enzyme–inorganic hybrid nanoflowers.

## Graphical abstract

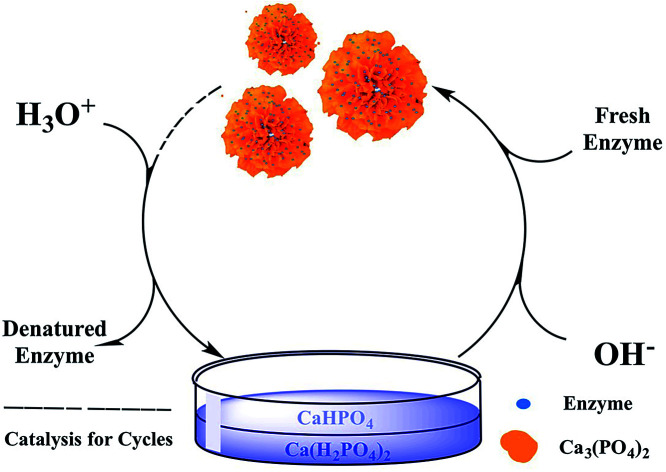

## 1. Introduction

Enzyme biocatalysis has been more and more widely used for its substrate specificity, low toxicity and the absence of production of undesirable products, which make enzymes have advantages compared with chemical catalysts in their catalytic reactions. However, low tolerance to extreme conditions such as high temperature, acidic or basic pHs and organic solvents and difficulty in separation from reaction mixture have been lying as a stumbling block in its industrial applications. Immobilization techniques could effectively overcome the above problems of free enzymes exactly to a great extent.^1–4^ Immobilized enzymes show improved stability, making them efficient,^5^ reusable and economical.^6^

In the enzyme immobilization, enzyme–inorganic hybrid nanoflowers as a new method has been attracting more and more attentions because of its simplicity and efficiency.^7–10^ Copper phosphate often was used as the inorganic component and enzyme as organics was confined in the inorganic salts crystals to form the hybrid nanoflowers.^11^ In the flowers, the enzymes usually present higher catalytic activity than that of corresponding free one. Nadar and his coworkers^12^ have prepared an organic–inorganic hybrid glucoamylase nanoflower and the hNFs exhibited 204% enhanced activity recovery. Yu *et al.*^13^ have synthesized organic–inorganic nanoflowers for crude soybean peroxidase (SBP) purification and exhibited 446% enhanced enzymatic activity. Although the Cu_3_(PO_4_)_2_·*x*H_2_O-hNFs had large superiority on activity and stability, in which the copper ion belong to heavy metal ion that could have extensive detrimental effects on humans and the environment due to their enhanced reactivity and high density.^14,15^ For example, copper ion induced metal lothioneins (MTs) through conjugation to “metallothionein-like protein” (MTLP).

On the contrary, calcium ion was the most biocompatible for its abundance in the bone of living organisms. There was also rich calcium ion in various foods such as milk, peanuts and beans. Thus, calcium ion is more environmentally friendly and greener than other metal ions in catalytic chemical reactions. Altinkaynak and his coworkers^16^ have ever introduced an elegant immobilization approach that it was discovered in synthesis of flower-like organic–inorganic hybrid nanostructures with extraordinary catalytic activity and stability. And it was demonstrated that the hybrid nanoflowers (hNFs) highly enhanced catalytic activities and stability in a wide range of experimental conditions. There were few reports that describe the hybrid nanoflowers using inorganic component of calcium phosphate. Ke and his coworkers^17^ have described a new hybrid nanoflower using the organic component of *Burkholderia cepacia* lipase and inorganic component of calcium phosphate and the activity was 308% folds of the free one. Yin *et al.*^18^ also have prepared hNFs using the calcium phosphate as the inorganic component and α-chymotrypsin (ChT) as the organic component. In addition, the hNFs can be used as an immobilized ChT reactor (IMER) for highly efficient protein digestion. Wang and his workers^19^ have reported a rational design of CaHPO_4_–α-amylase hybrid nanobiocatalytic system based on allosteric effect and an explanation of the increase in catalytic activity when certain enzymes are immobilized in specific nanomaterials. The hNFs exhibited dramatically enhanced enzymatic activity due to the allosteric modulation and its hierarchical structure.

However, much attentions were paid to the reuse of enzyme and little was paid to that of support in enzyme immobilization. When the immobilized enzymes lost their catalytic activity, they were abandoned after some recycles, which also resulted in the pollution and increase of the cost of the immobilized enzyme. For example, superabundant calcium and phosphate ions were also harmful and unwelcome to environment,^20,21^ especially in the water and soil. In this work, the reuse of six kinds of enzymes were achieved by the assembly of enzyme–inorganic hybrid nanoflowers, but also the inorganic supports were recycled when the hNFs lost their activity by calcium phosphate dissolution and recrystallization. The catalytic properties of the obtained hNFs were examined and the difference of the activities and morphology of the original and dual-cycled enzyme–inorganic hNFs were investigated.

## 2. Materials and methods

### 2.1. Materials

Papain and bromelain were purchased from shanghai Tripod Biological Technology Ltd. CALB and TLL were purchased from Novozymes. PPL was purchased from Tokyo Chemical Industry (TCI). *N*-Benzoyl-l-arginine ethyl ester hydrochloride (BAEE) were purchased from Aladdin. 4-Nitro phenol was purchased from sigma. Benzoyl acetone and 4-nitrophenyl acetate were purchased from Alfa Aesar chemistry Ltd. (Tianjin). All other chemicals were provided by Sinopharm Chemical Reagent Ltd. (Shanghai).

### 2.2. Preparation of original enzyme–Ca_3_(PO_4_)_2_ hybrid nanoflowers

**Fig. 1:**
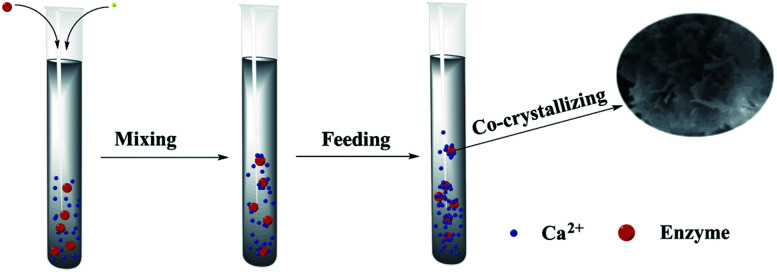
Scheme for preparing enzyme–Ca_3_(PO_4_)_2_ hybrid nanoflowers crystal.

The hNFs were synthesized in a typical experiment: 100 μL CaCl_2_ (200 mM) was added to 5 mL of a phosphate buffered saline (PBS) solution (20 mM) containing different concentration enzyme at pH 6.7, and the mixture was kept at 4 °C for 24 h. In the following experiments, the conditions were kept the same as the typical experiment, unless stated otherwise. The mixture was centrifuged at 4 °C and 10 000 rpm for 5 min to remove the supernatant and obtain the enzyme–Ca_3_(PO_4_)_2_-hNFs (Fig. 1).

### 2.3. Dual-cycle for enzyme and calcium phosphate support

**Fig. 2:**
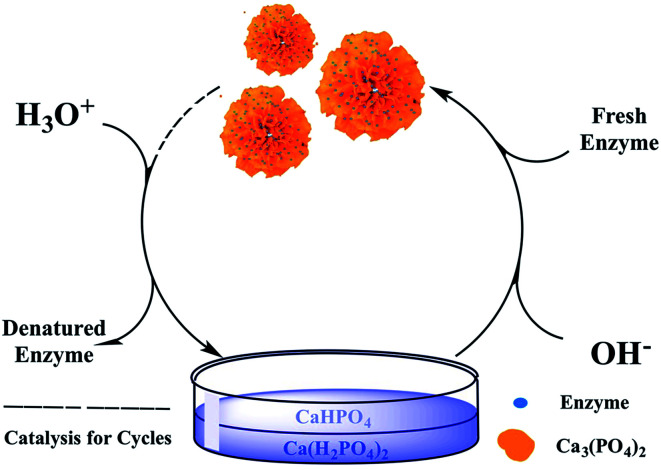
The process for achieving the cleaner and dual-cycle immobilization for enzyme and support.

The process of dual-cycle for enzyme was shown in Fig. 2. 0.2 mL acids such as phosphoric acid or acetic acid were added into the used original enzyme-hNFs to dissolve them. Then the solution was heated at 100 °C for 10 min to denature enzymes proteins which were removed by centrifugation or filtration. The pH of the cooled supernatant or filtrate was adjusted to 6.7 using Ca(OH)_2_ aqueous. Fresh enzymes were then mixed with the solution and co-crystallized with Ca_3_(PO_4_)_2_ at 4 °C for 24 h to form the rebloomed hNFs. They were separated as the above method and procedure and used to catalyze the hydrolytic and synthetic reactions as the original hNFs.

### 2.4. Enzyme–inorganic hybrid nanoflowers activity assay

Lipase activity was determined according to the method reported by Cheong,^22^ the modified procedure was as followed: enzyme solution (25 μL) was transferred to 1.4 mL of the PBS (100 mM, pH 6.5). The solution was warmed for 3 min at 37 °C and 150 rpm. Then added *p*-nitrophenol acetate (75 μL, 50 mM) and shook for 3 min. At the last, the absorbance of the solution after adding acetone (1.5 mL) was measured at 405 nm. When the enzyme was the protease, the way was as followed:^23^ enzyme solution (100 μL) was placed in water bath at 37 °C for 5 min. Then added Na-benzoyl-l-arginine ethyl ester hydrochloride (1 mL) and reacted for 10 min. The absorbance of the solution was measured at 253 nm.

### 2.5. Characterization of enzyme–inorganic hybrid nanoflowers and their catalytic synthesized products

Scanning electron microscope (SEM) (S-4800, HITACHI) was employed to examine the morphology of hNFs and Gas Chromatography Mass Spectrometer (GC-MS, Agilent 5975) was used to measure the molecular weight of products. ^1^H NMR spectra and ^13^C NMR were recorded through a Brucker DPX 300 spectrometer at 500 MHz and 126 MHz, respectively. NMR experiment was performed in deuterated chloroform (CDCl_3_) containing proper concentration of sample, a total 128 scans were recorded. Data was collected and analyzed through MestReNova software. Proton chemical shifts were referenced to tetramethylsilane (TMS) at 0.00 pm.

## 3. Results and discussions

### 3.1. Characterization of enzyme–inorganic hybrid nanoflowers (hNFs) preparations

**Fig. 3:**
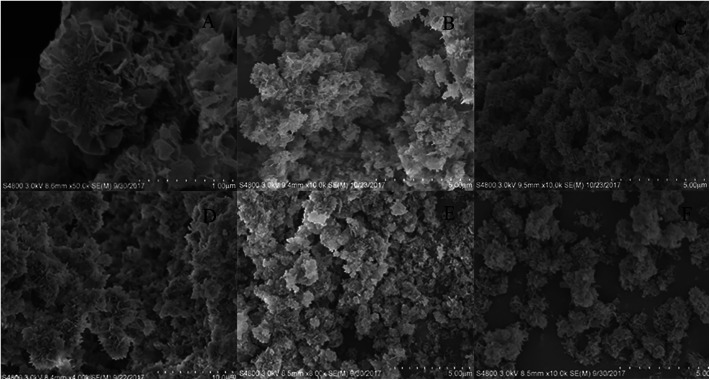
SEM images of the hNFs of different enzymes ((A) papain; (B) bromelain; (C) trypsin; (D) TLL; (E) CALB; (F) PPL).

The samples were synthesized using PBS (4 mM, pH 7.4) containing 0.1 mg mL^−1^ of enzyme at 4 °C for 12 h and SEM was used to examine the morphology and structure of the obtained enzyme–inorganic hNFs. It was found that the follower-shape of Ca_3_(PO_4_)_2_-hNFs was not obvious as Cu_3_(PO_4_)_2_-hNFs,^9^ which may due to the different inorganic salt crystals for crystal copper phosphate and amorphous calcium phosphate. As show in Fig. 3, the SEM image of the lipase-hNFs (Fig. 3D–F) had the flower-shape and it was better than the hNFs that has been reported before.^17^ The results displayed that the morphology of the lipase-hNFs was more like flower than protease-hNFs (Fig. 3A–C). Besides, the pictures of the TLL-hNFs were clearest and the petals can be seen more obviously. The SEM images of hNFs containing different enzyme were different, which probably attributed to the different coordination ability between Ca^2+^ with protein according to the initiation of the forming process of hNFs by Ge.^7^

### 3.2. Catalytic activity of enzyme–inorganic hybrid nanoflowers preparations

**Fig. 4:**
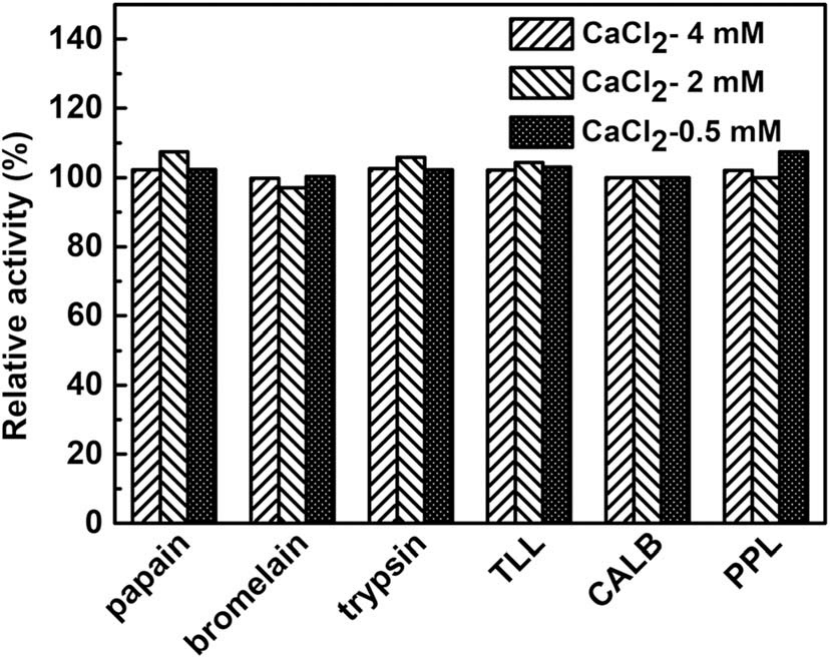
Effect of calcium ion and its concentration on enzymatic activity.

To examine the effect of calcium ion on the enzymatic activity and avoid the its inhibition on the hNFs activity, the used six enzymes all undergo the catalysis in reaction mixture containing three kinds of concentration (0.5 mM, 2 mM, 4 mM) of calcium ion. As shows in Fig. 4, at the three concentration level (0.5 mM, 2 mM, 4 mM) of calcium ion, no clear effect was observed in the effect of the activity of six used model enzymes. In fact, more or less calcium ion often plays a positive role in enzymatic activity for different enzymes. For example, Ca^2+^ has increasing effect on the enzymatic activity for chondroitinase ABC I^24^ and an adequate concentration of CaCl_2_ was crucial to maintain the enzymatic activity of salivary α-amylase.^25^

**Fig. 5:**
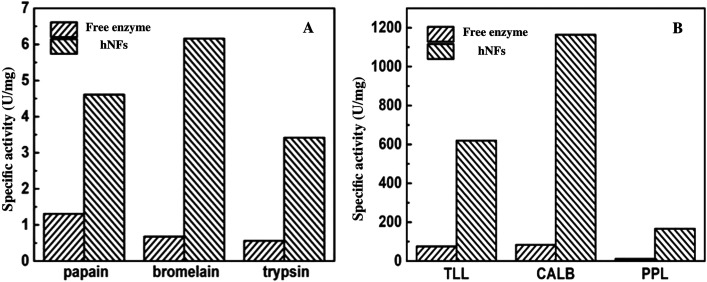
Comparison of catalytic activity between free enzyme with hNFs. (A) Protease; (B) lipase.

Fig. 5 described the comparison of the activities between free enzymes with hNFs. The results show that the activities of hNFs were better than that of free enzymes and the activities were all improved by nearly ten folds after being confined in hybrid nanoflowers for enzymes. The maximum hydrolytic activity of CALB-hNFs was up to 14.0 folds of that of free one. It was obvious that the enhancement effect of lipase-hNFs in activity was better than that of protease-hNFs, which may result from the morphology and structure of enzyme–inorganic hybrid nanoflowers.

### 3.3. Comparison of activity between the original and the dual-cycled hNFs

**Fig. 6:**
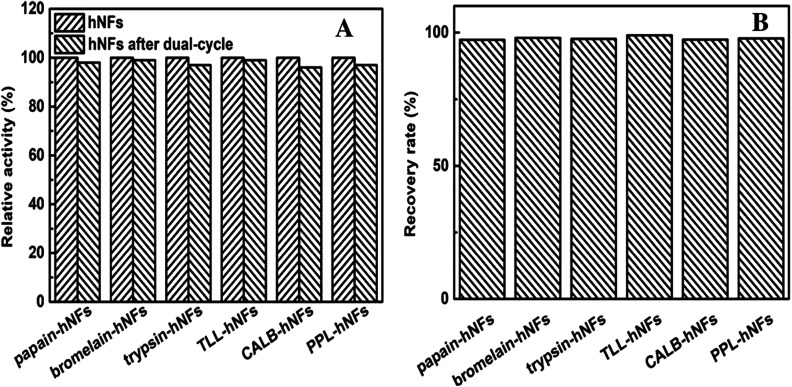
Comparison between the hNFs and hNFs after dual-cycle (A) relative activity of hNFs and the hNFs after dual-cycle; (B) recovery rate of Ca_3_(PO_4_)_2_ after dual-cycle.

When the original hNFs lost their activity, they were used to prepare the dual-cycled hNFs by hNFs dissolution and recrystallization together with fresh enzymes to recycle the calcium phosphate. The activities of the dual-cycled hNFs were investigated and the recovery of the calcium phosphate was examined carefully. The results in the Fig. 6A show that there was no obvious difference in relative activity between dual-cycled hNFs and original ones, which means that some molecules such as amino acids didn't exert influence on the catalysis of the dual-cycled hNFs. This may result from the good biocompatibility of these molecules lysed from the used enzymes protein in the recovery of the calcium phosphate. The results in Fig. 6B shows the recovery rate of the phosphate calcium by weight-checking after their recrystallization and dry. It was found that the recovery rate of Ca_3_(PO_4_)_2_ were all above 95%, even up to the 99% for the six enzyme–inorganic hybrid nanoflowers. On the whole, the activities of the dual-cycled hNFs and the weight of Ca_3_(PO_4_)_2_ were almost constant and invariable for the six model enzymes hNFs after dual-cycle. In addition, to investigate the leaching of the enzyme protein from hNFs, we examined free enzyme protein content in reaction mixture by Bradford method and no protein was detected. However, the enzyme hNFs were recovered by centrifuging and would lose more or less due to its small size.

### 3.4. Thermal stability of enzyme–inorganic hybrid nanoflowers preparations

**Fig. 7:**
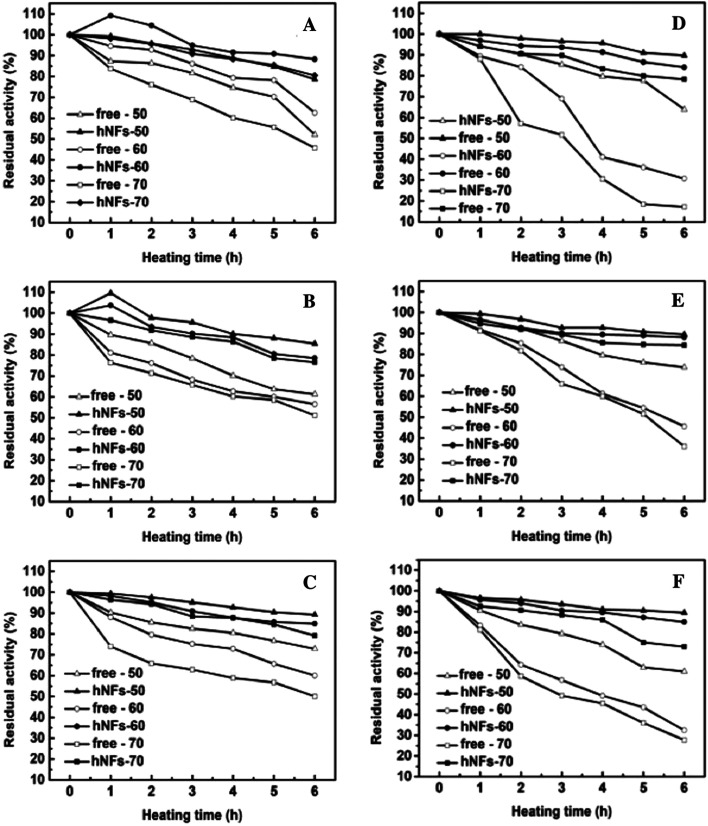
Thermal stability of free enzyme and enzyme-hNFs preparations ((A) papain; (B) bromelain; (C) trypsin; (D) TLL; (E) CALB; (F) PPL).

The comparison between free enzyme and hNFs in thermal stability were investigated under three temperatures conditions (50 °C, 60 °C, 70 °C) and the results were shown in the Fig. 7. It was obvious that the thermal stabilities of all the obtained hNFs were better than the corresponding free enzymes, respectively. Even though the thermal stabilities of free protease were better than that of lipase, the immobilization give much clearer enhancement in thermal stability of lipase than that of protease. After heating for 6 h at 70 °C, the residual activities of TLL-hNFs, PPL-hNFs, and CALB-hNFs, were 78.3%, 72.9% and 84.3%, which were 4.57, 2.61 2.35 folds of that of their corresponding free one. However, the most enhancement in thermal stabilities for protease in our work was achieved for papain-hNFs and the enhancement times was only 1.8. Moreover, when CALB was immobilized on a macroporous polyacrylate carrier including Novozym 435, the residual activity was about 20% after heating at 70 °C.^26^ The high enhancement in thermal stabilities for enzyme-hNFs possible resulted from the strong interaction of much Ca^2+^ ions and the functional groups of enzyme protein^27–29^ in the hybrid nanoflowers. The strong interaction and the rigidity in the inorganic hNFs confined the enzyme protein structure and prevented the peptide chains from unfolding and improved the enzyme thermal stability.

### 3.5. Kinetics determination about free enzyme and hNFs

The related kinetic parameters such as *K*_m_, *K*_cat_, *K*_m_/*K*_cat_ were investigated and calculated according to the results of the activity experiments and listed in Table 1. It was found from Table 1 that the *K*_m_ values of free enzymes was all higher than that of the corresponding hNFs, which mean that the hNFs had a better affinity for their substrates because *K*_m_ value could approximately reflect the affinity of substrate to enzyme.^30^ What's more, the *V*_max_ values of free enzyme were higher than that of protease-hNFs, while it was just reverse for lipase from the Table 1. Protease-hNFs showed lower *V*_max_ and *K*_cat_ values than free one, which suggested a reduced product release velocity, which appeared to be in accordance with the findings described by Benucci.^31^ However, it was worthy pointing out that the highest catalytic efficiency (*K*_cat_/*K*_m_) of TLL-hNFs was 38.52 mM^−1^ s^−1^, 21.7 folds than that of free enzyme. In addition, the enhancement in the catalytic efficiency was derived from the facile immobilization by directly forming the enzyme-hNFs without further chemical modification of enzyme.^32^ The chemical modification often decreased the catalytic activity of enzyme when other molecules were coupled to the functional groups of side chain of enzyme structure.

**Table 1:** The kinetics parameters of free enzyme and hNFs preparations

Enzyme preparations	*K*_m_ (mM)	*K*_cat_ (s^−1^)	*K*_cat_/*K*_m_ (mM^−1^ s^−1^)
Papain	1.01 ± 0.05	2.67 ± 0.01	2.67 ± 0.125
Papain-hNFs	0.7 ± 0.025	1.87 ± 0.1	2.65 ± 0.452
Bromelain	0.93 ± 0.04	0.38 ± 0.02	0.4 ± 0.005
Bromelain-hNFs	0.46 ± 0.02	0.19 ± 0.01	0.4 ± 0.13
Trypsin	0.43 ± 0.022	0.5 ± 0.026	1.17 ± 0.001
Trypsin-hNFs	0.07 ± 0.0029	0.09 ± 0.004	1.27 ± 0.004
TLL	16.58 ± 0.793	29.3 ± 1.265	1.77 ± 0.008
TLL-hNFs	0.17 ± 0.009	6.55 ± 0.288	38.52 ± 0.347
CALB	6.36 ± 0.321	56.76 ± 2.732	8.92 ± 0.023
CALB-hNFs	0.14 ± 0.007	6.14 ± 0.158	42.91 ± 1.078
PPL	0.9 ± 0.046	4.44 ± 0.212	4.93 ± 0.025
PPL-hNFs	0.09 ± 0.003	5.96 ± 0.278	63.36 ± 0.88

### 3.6. Enzyme-hNFs catalysis in the chemical synthesis

**Fig. 8:**
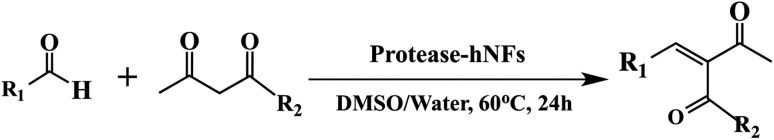
Protease-hNFs catalytic Knoevenagel condensation.

To further examine the catalytic properties of the obtained enzyme-hNFs in chemical synthesis, the protease-hNFs were used to catalyze the promiscuous catalytic Knoevenagel condensation and lipase-hNFs were used to catalyze the clindamycin palmitate synthesis. For protease-hNFs, they also could catalyze the knoevenagel condensation using benzaldehyde and acetylacetone as substrates as the report^33^ (Fig. 8). The yield of product (Table 2) using papain-hNFs was 28.2%, similar to that using bromelain-hNFs and trypsin-hNFs. The characterization of the product was showed in the ESI (see Fig. S1–S3†).

**Table 2:** Synthesis yield of product in Knoevenagel condensation using protease-hNFs as catalysts^a^

Entry	Papain-hNFs	Bromelain-hNFs	Trypsin-hNFs
Yield (%)^b^	28.2	23.6	25.4

a Conditions: benzaldehyde (2 mmol), acetylacetone (2.4 mmol), hNFs (150 mg), DI water (1.25 mL), DMSO (3.75 mL) at 60 °C for 24 h.

b Yield of the isolated product after purification.

**Fig. 9:**
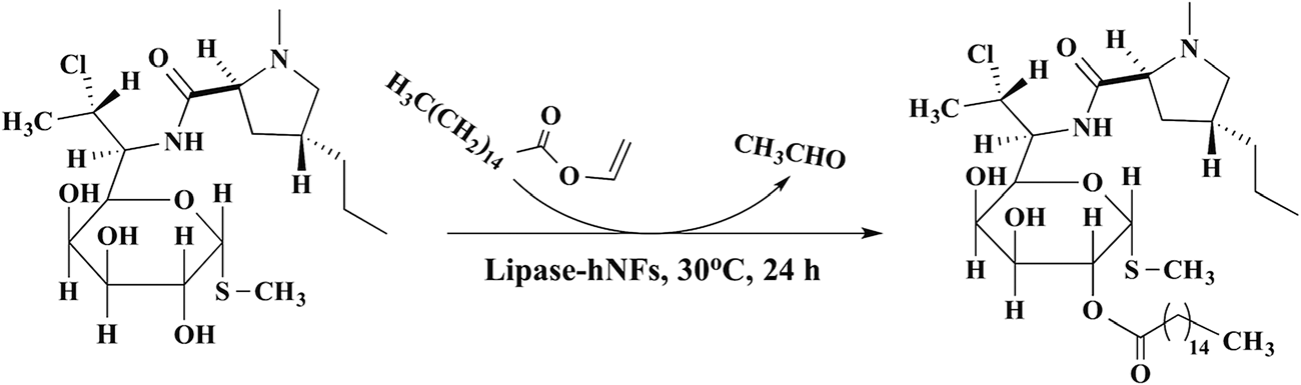
Lipase-hNFs catalytic synthesis of clindamycin palmitate.

For lipase-hNFs catalysis in the synthesis of clindamycin palmitate (Fig. 9), the yield was up to 71.9% when TLL-hNFs was used as catalyst, higher by 1.47 times than that using CALB-hNFs. For PPL-hNFs, it seemed to present no catalytic ability in the synthesis of clindamycin palmitate. When compared with the standard commercial immobilized enzyme in the enzymatic synthesis of clindamycin palmitate, the yield of clindamycin palmitate catalyzed by commercial immobilized TLL (named Lipozyme TL IM) was only 57% which was also less than that obtained using the TLL@Ca_3_(PO_4_)_2_ hNFs. The characterization of the product was show in the ESI (see Fig. S4–S7†) (Table 3).

**Table 3:** Synthesis yield of clindamycin palmitate using lipase-hNFs as catalysts^a^

Entry	TLL-hNFs	CALB-hNFs	PPL-hNFs
Yield (%)^b^	71.9	49.1	1.6

a Conditions: clindamycin free base (0.3 mmol), vinyl palmitate (0.9 mmol), petroleum ether (6.25 mL) at 30 °C for 24 h.

b Yield of the product detected by HPLC.

## 4. Conclusion

In conclusion, we reported a cleaner and dual-cycle immobilization for enzyme and support by reblossoming enzyme–inorganic hybrid nanoflowers. At first, we prepared the enzyme co-embedded organic–inorganic hybrid nanoflowers by using calcium phosphate as the inorganic component and 6 enzymes (papain, bromelain, trypsin, TLL, CALB and PPL) as the organic components, respectively. When the obtained original enzyme-hNFs lost their activity after reuse for many cycles, the inorganic component in the hNFs was recovered by acidification using phosphoric acid or acetic acid specially derived from biomass to achieve the reuse of support in enzyme immobilization. We hope that our work will contribute to a further understanding of the complicated enzymatic reactions and further enrich the enzyme immobilization research.

## Conflicts of interest

There are no conflicts to declare.

## Supplementary Material

RA-008-C8RA02051E-s001
