# Supplementary material for: Dual-cycle immobilization to reuse both enzyme and support by reblossoming enzyme–inorganic hybrid nanoflowers
Source: RSC Adv. 2018 Apr 30;8(29):16088–94. doi: 10.1039/c8ra02051e (PMC9080259; doi:10.1039/c8ra02051e)
Supplement: RA-008-C8RA02051E-s001 [file RA-008-C8RA02051E-s001.pdf]

Electronic Supplementary Material (ESI) for RSC Advances.
This journal is © The Royal Society of Chemistry 2018

Supplementary information

Dual-cycle immobilization to reuse both enzyme and support by reblossoming enzyme-inorganic hybrid nanoflowers

[a]Jianyun Yu, [a]Chenhui Wang, [a]Anming Wang*, [a]Ningning Li,
[a]Xinxin Chen, [a]Xiaolin Pei*, [a]Pengfei Zhang, [b]Stephen Gang Wu

[a]College of Materials, Chemistry and Chemical Engineering,
Hangzhou Normal University, Hangzhou 310014, P. R. China
[b]Department of Energy, Environmental and Chemical Engineering,
Washington University, St. Louis, MO 63130, USA

The characterization of products synthesized using enzyme-hNFs catalysts

MS, ^{1}HNMR and ^{13}CNMR were used to characterize the obtained products, respectively. Fig.S1 described the MS of 3-benzylidenepentane-2,4-dione catalyzed by hNFs in DMSO containing 25% water for 24 h. The molecular weight of the product was 188.23 and the ion peak was 189.07 shown in S1. The ion peak should be the M+H peak, so the result conformed to the molecular weight of the product.

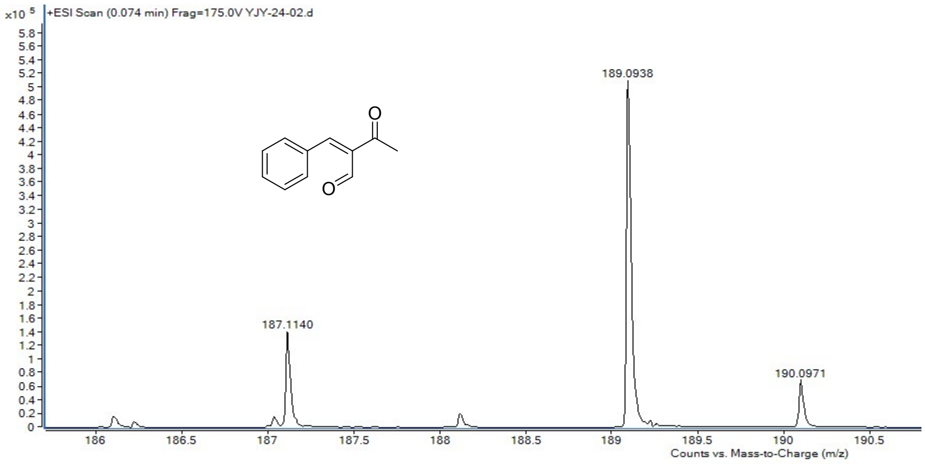

Figure S1 the MS of 3-benzylidenepentane-2,4-dione

The ^{1}HNMR spectrum of 3-benzylidenepentane-2,4-dione catalyzed by hNFs in DMSO containing 25% water for 24h, along with peak positions and assignments, was displayed in Fig.S2. The data were as followed: δ 7.41 (s, 1H), 7.31 (m, 5H), 2.34 (s, 3H), 2.20 (s, 3H).

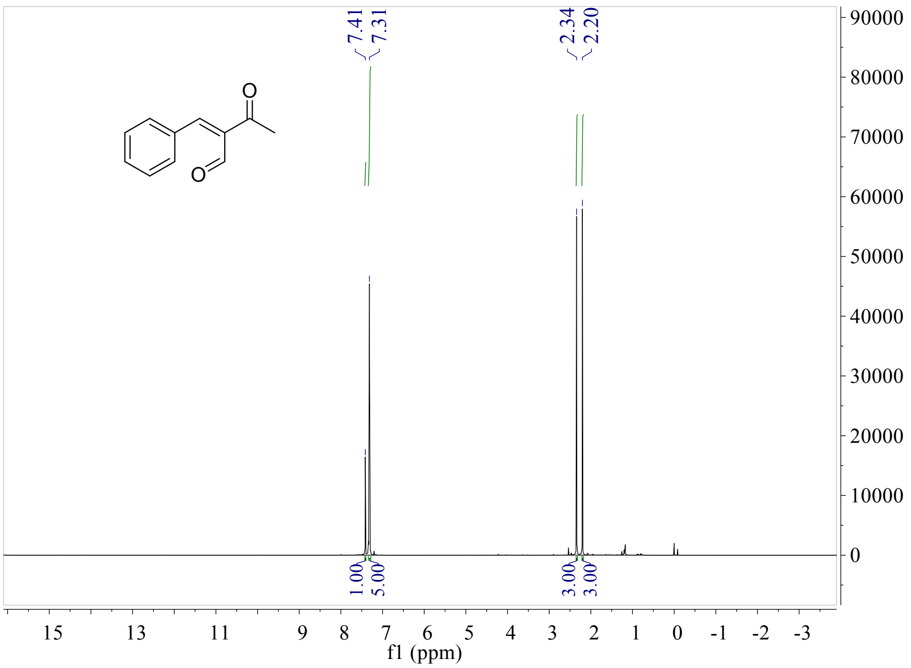

Figure S2 ^{1}HNMR of 3-benzylidenepentane-2,4-dione

The ^{13}CNMR spectrum of 3-benzylidenepentane-2,4-dione catalyzed by hNFs in DMSO containing 25% water for 24h, along with peak positions and assignments, was displayed in Fig.S3. The data were as followed: δ 204.57 (s), 195.53 (s), 141.77 (s), 138.82 (s), 131.87 (s), 129.65 (s), 128.68 (s), 128.02 (s), 30.62 (s), 25.47 (s).

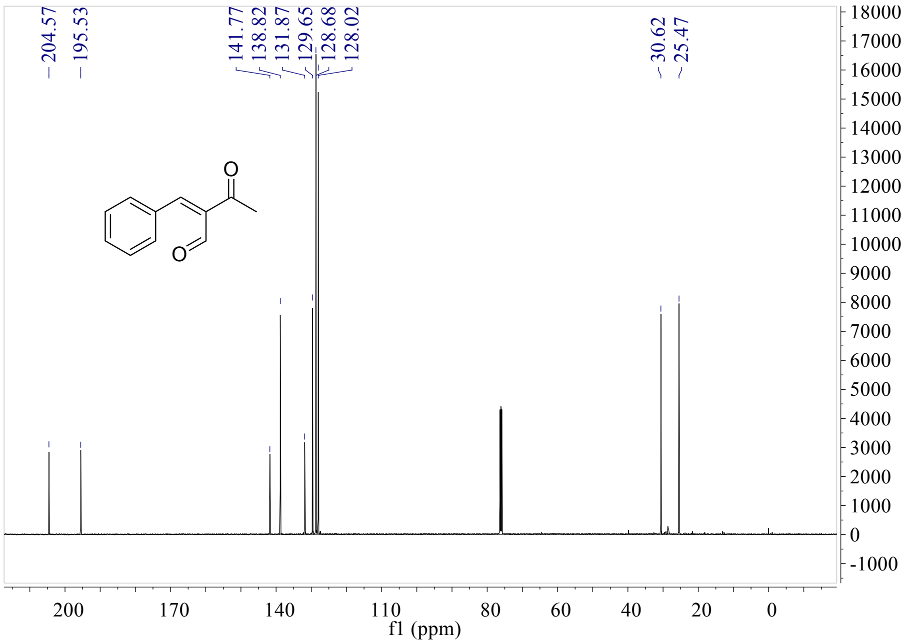

Figure S3 ^{13}CNMR of 3-benzylidenepentane-2, 4-dione

MS, ^{1}HNMR and ^{13}CNMR were used to characterize the obtained products, respectively. Fig.S4 described the MS of clindamycin palmitate catalyzed by hNFs in PE for 24 h. The molecular weight of clindamycin palmitate was 663.4 and the ion peak was 663.9 shown in Fig.S4. The ion peak should be the M+H peak, so the results conformed to the molecular weight of clindamycin palmitate.

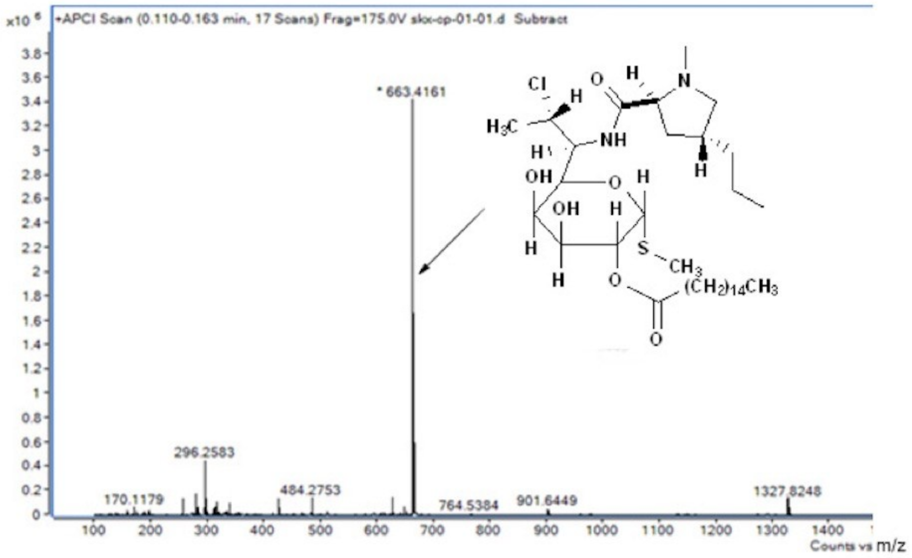

Figure S4 the MS of the clindamycin palmitate

The ^{1}HNMR spectrum of clindamycin palmitate catalyzed in PE for 24 h, along with peak positions and assignments, was displayed in Fig.S5. The data were as followed: ^{1}H NMR (500 MHz, DMSO) δ 9.83 (s, 1H), 8.75 (t, J = 26.0 Hz, 1H), 5.42 (d, J = 5.6 Hz, 1H), 5.00 (dd, J = 10.4, 5.6 Hz, 1H), 4.53 – 4.43 (m, 2H), 4.23 (d, J = 8.0 Hz, 1H), 4.14 (d, J = 9.9 Hz, 1H), 3.87 (s, 1H), 3.68 – 3.57 (m, 2H), 3.35 (s, 4H), 2.85 (s, 3H), 2.55 – 2.46 (m, 4H), 2.29 (t, J = 7.3 Hz, 4H), 2.06 (s, 4H), 1.54 (s, 2H), 1.41 (d, J = 7.1 Hz, 2H), 1.35 (s, 2H), 1.30 – 1.19 (m, 31H)

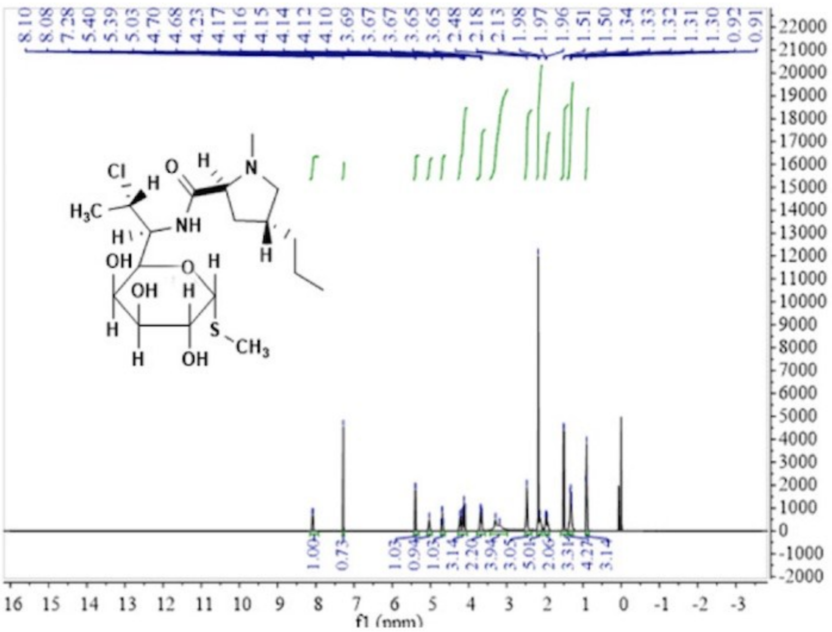

Figure S5 the ^{1}HNMR of the clindamycin palmitate

The ^{13}CNMR spectrum of clindamycin palmitate catalyzed in PE for 24 h, along with peak positions and assignments, was displayed in Fig.S6. The data were as followed: ^{13}C NMR (126 MHz, DMSO) δ 170.60 (d, J = 639.7 Hz), 84.35 (s), 75.12 – 63.38 (m), 60.17 (d, J = 114.9 Hz), 52.98 (s), 35.17 (dd, J = 184.5, 81.4 Hz), 31.77 (s), 30.27 – 27.50 (m), 22.85 (dd, J = 279.1, 212.8 Hz), 16.41 – 10.76 (m).

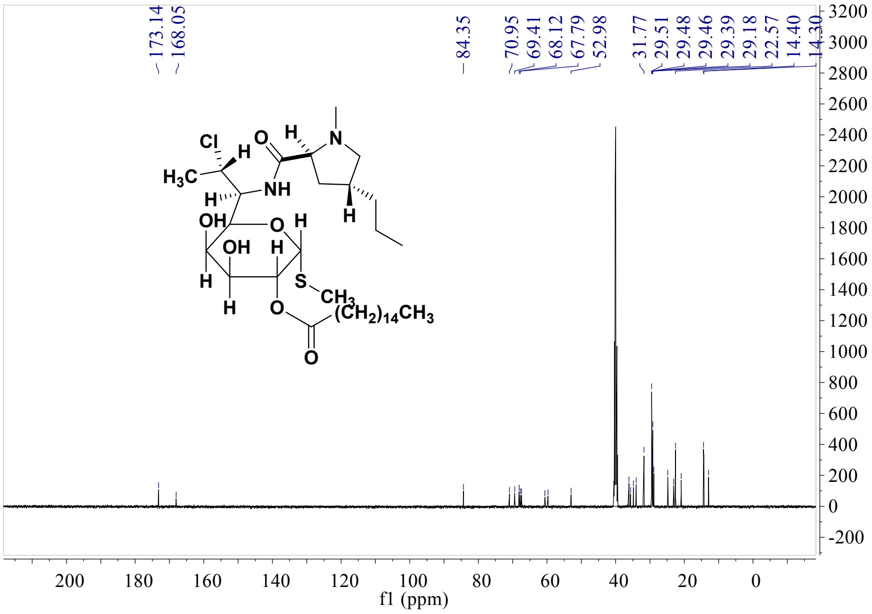

Figure S6 the ^{13}CNMR of the clindamycin palmitate

The conversions of clindamycin palmitate were analyzed by HPLC, using a mixture of methanol and 3% ammonium acetate water solution (70:30， v/v) as the mobile phase at a flow rate of 0.5 mL/min. The temperature of column oven was maintained at 25℃. UV detection was carried out at 226 nm. The retention times for clindamycin, vinyl palmitate and clindamycin palmitate were at 6.23, 11.383 and 28.427 mins, respectively (Fig. S7).

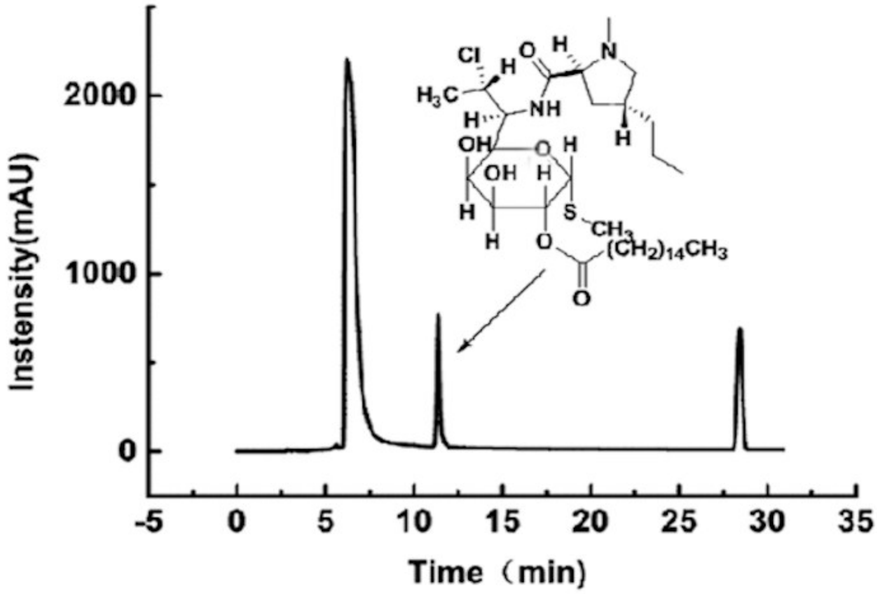

Figure S7 the HPLC result of the synthetic reaction catalyzed by hNFs in PE for 24 h
